# Intraoperative Radiation Therapy for Recurrent Cervical and Endometrial Cancer: Predicting Morbidity and Mortality in a Contemporary Cohort

**DOI:** 10.3390/cancers16213628

**Published:** 2024-10-28

**Authors:** Lindsay N. Howlett, Priyal P. Fadadu, Leah O. Grcevich, Angela J. Fought, Michaela E. McGree, Andrea Giannini, Kristina A. Butler, Lucia Tortorella, Amanda A. Marnholtz, Michael G. Haddock, Allison E. Garda, Carrie L. Langstraat, Sean C. Dowdy, Amanika Kumar

**Affiliations:** 1 Alix School of Medicine, Mayo Clinic, 200 First St. SW, Rochester, MN 55905, USA; howlett.lindsay@mayo.edu; 2 Department of Obstetrics and Gynecology, Mayo Clinic, 200 First St. SW, Rochester, MN 55905, USA; 3 Department of Biostatistics, Mayo Clinic, 200 First St. SW, Rochester, MN 55905, USA; 4 Department of Gynecologic Oncology, Mayo Clinic, 13400 East Shea Blvd., Scottsdale, AZ 85259, USA; 5 Department of Women’s Health, Children’s Health and Public Health, Agostino Gemelli University Polyclinic (IRCCS), Largo Agostino Gemelli, 8, 00136 Rome, Italy; 6 Department of Radiation Oncology, Mayo Clinic, 200 First St. SW, Rochester, MN 55905, USA; 7 Department of Gynecologic Oncology, Mayo Clinic, 200 First St. SW, Rochester, MN 55905, USA

**Keywords:** intraoperative radiation therapy, endometrial cancer, cervical cancer, recurrent, persistent, gynecologic cancer

## Abstract

Background/Objectives: Our objective was to describe the use of intraoperative radiation therapy (IORT) for the treatment of recurrent/persistent cervical or endometrial cancer and assess predictors of postoperative complications and 3-year mortality. Methods: In this multi-site retrospective study, data were abstracted for recurrent/persistent endometrial or cervical cancer patients who underwent IORT from June 2004 to May 2021. Complications were graded on the six-point Accordion scale. Variables associated with complications were analyzed with univariate logistic regression, while variables associated with death within 3 years were analyzed with Cox proportional hazards modeling. Survival was analyzed with the Kaplan–Meier method. Results: Eighty patients had planned IORT for recurrent/persistent endometrial (n = 35) or cervical cancer (n = 45). The mean age of the cohort was 56.8 years (SD = 13.7), and the median disease-free interval from primary disease to recurrence was 20.0 months (IQR 10.0–63.1). The overall survival at 3 years was 48.6% (95% CI: 38.3–61.6%) with a median survival of 2.8 years. Within 30 days postoperative, 16 patients (20.1%) had grade 3–5 complications and one death (1.3%) occurred. Factors associated with grade 3+ complication included ECOG PS 2–3 (OR 18.00, *p* = 0.04), neoadjuvant chemotherapy and/or immunotherapy (OR 6.98, *p* < 0.01), and pelvic sidewall involvement (OR 8.80, *p* = 0.04). Factors associated with death within 3 years of surgery included ECOG PS 2–3 (HR 8.97, *p* < 0.01), neoadjuvant chemotherapy and/or immunotherapy (HR 2.34, *p* = 0.03), whether exenteration was performed (HR 2.64, *p* = 0.01), and positive resection margin (HR 3.37, *p* < 0.01). Conclusions: In well-selected patients, IORT is a feasible and safe option for the treatment of recurrent/persistent gynecologic malignancy with an appreciable survival benefit.

## Simple Summary

Intraoperative radiation therapy (IORT) can be a useful treatment modality in patients with recurrent cervical or endometrial cancer. The existing literature on IORT use in recurrent gynecologic malignancies is limited by small sample sizes because few centers use IORT and few patients are ideal candidates. The aim of our study is to describe a modern cohort of patients who were considered for IORT and predict risk factors of morbidity and mortality. We found that appreciable survival gain can be achieved with the use of IORT, with factors such as ECOG performance status (ECOG PS), neoadjuvant chemotherapy/immunotherapy, pelvic sidewall involvement, whether exenteration was performed, and resection margin status influencing the risk of morbidity and/or mortality.

## 1. Introduction

In 2024, there will be an estimated 82,000 new cases of cervical or endometrial cancer diagnosed in the United States and 18,000 deaths from these malignancies [1]. Though initial treatment via surgery, radiation (RT), and/or chemotherapy can be curative, recurrence rates range from 4 to 20% for endometrial cancer and 25 to 61% for cervical cancer [2,3,4]. In patients experiencing recurrence, the success of treatment depends heavily on the location of the recurrence, prior treatment type, and timing of recurrence. Specifically, the majority of cases are distant and/or multi-site and, therefore, require palliative systemic treatment approaches with chemotherapy, immunotherapy, or targeted agents. In cases of local recurrence of endometrial or cervical cancer, salvage RT or repeat surgical resection can be curative [5,6,7,8,9,10].

In the unique case of isolated recurrence in the pelvis or retroperitoneal lymph nodes, radical oncologic resection obtaining negative margins can be a curative intent approach [11,12,13]. The addition of intraoperative radiation therapy (IORT) to radical oncologic resection may improve local control and has been used in the treatment of gynecologic, neurologic, colorectal, genitourinary, and pancreatic malignancies [14,15,16,17,18,19,20,21]. In the setting of previously irradiated fields, IORT offers the ability to target the field at the highest risk of recurrence while sparing healthy tissues [22]. It is estimated that this larger, single, and more targeted radiation dose is 2–3 times as effective as the same dose delivered in fractionated external beam radiation therapy (EBRT) doses [23]. In general, IORT is used in patients who are fit for radical surgery, do not have distant metastases, and are at higher risk of recurrence and/or have close or positive resection margins. From a gynecologic perspective, it can be particularly beneficial on the pelvic sidewall, lymph node basins, and along bony structures where the morbidity of expanded wider surgical margins would be significant. Additionally, sidewall involvement has traditionally been considered a contra-indication for surgical resection in recurrent gynecologic cancer; however, our experience is that with the use of RT, we can provide a meaningful resection.

Prior studies on the use of IORT for recurrent cervical and endometrial cancer have demonstrated 5-year overall survival ranging from 14% to 47% [14,15,23,24]. The existing literature is limited by small sample sizes of patients who have undergone IORT because few centers utilize IORT for gynecologic cancer and few patients are ideal candidates. Short-term morbidity associated with surgery combined with IORT is rarely discussed in the existing reports of IORT outcomes. To address these gaps in the literature, our multi-site retrospective cohort study of recurrent cervical and endometrial cancer patients undergoing planned IORT at the time of radical oncologic resection updates the existing knowledge with a contemporary cohort. By addressing both the potential survival benefit and complications associated with this procedure, we aim to better understand which patients are most likely to benefit from this treatment modality.

## 2. Materials and Methods

This is a multi-site retrospective cohort study of patients who had a treatment plan of radical resection with IORT for recurrent/persistent endometrial or cervical cancer at Mayo Clinic in Minnesota, Arizona, or Florida from June 2004 to May 2021 and was approved by the Institutional Review Board. The inclusion criteria were diagnosis of recurrent or persistent malignancy with a primary site of the endometrium or cervix. Sarcomas (leiomyosarcoma and endometrial stromal sarcoma) and non-endometrioid histology (serous, clear cell, and carcinosarcoma) were excluded. Patient characteristics, including age, body mass index (BMI), Eastern Cooperative Oncology Group performance status (ECOG PS), smoking status, and medical comorbidities, were abstracted from patient records. The collected medical comorbidities included history of myocardial infarction, congestive heart failure, percutaneous coronary intervention (PCI) or prior cardiac surgery, hypertension requiring medication, diabetes mellitus, chronic obstructive pulmonary disease (COPD), transient ischemic attack (TIA) or cerebrovascular accident (CVA), venous thromboembolism (VTE), depression, renal failure, liver disease, autoimmune disease, rheumatologic disease, or hyperlipidemia.

The evaluated disease characteristics included histology, prior oncologic treatment of the disease, disease-free interval, neoadjuvant treatment (chemotherapy, immunotherapy, or radiation) after a diagnosis of recurrence and prior to surgery involving IORT, timing of disease as persistent or recurrent, and location of the recurrence. The disease-free interval was defined as the time between the end date of the last oncologic treatment and the date of recurrence/persistence diagnosis. Persistent cases were defined by radiological persistence or recurrence of disease within 6 months from completion of primary treatment and were excluded from the calculation of the disease-free interval. Prior oncologic treatment of the disease included radiation therapy involving brachytherapy and/or EBRT, as well as prior surgical treatment and/or systemic chemotherapy. Radiation-sensitizing chemotherapy was not considered systemic chemotherapy for the purpose of this analysis.

Intraoperative information was collected, including whether IORT was delivered as planned, whether concurrent pelvic exenteration was performed, and the type and extent of exenteration (anterior, posterior, or total) if performed. In the cases in which IORT was planned but not given, information from the operative reports was collected regarding the reasoning for excluding IORT. All additional surgical procedures were documented. Estimated blood loss and intraoperative transfusion were recorded. Pathologic data collected consisted of resection margin and tumor diameter. Postoperative complications occurring within 30 days postoperatively were graded on the expanded 6-point Accordion grading system, with severe complications receiving a grade of 3 or more. Accordion grade 3 complications are those requiring management by an endoscopic, interventional procedure, or reoperation without general anesthesia. Grade 4 complications require management by an operation under general anesthesia, while grade 5 complications involve organ system failure. Postoperative death is defined as a grade 6 complication [25].

### Statistical Analysis

Continuous variables were described by either the mean and standard deviation (SD) or by the median and interquartile range (IQR). Categorical variables were described by frequency and percentage of the cohort. Univariate logistic regression was used to assess associations with Accordion grade 3+ complications. Associations were summarized with odds ratios (ORs) and 95% confidence intervals (CIs). Overall survival was analyzed with the Kaplan–Meier method. Univariate analysis of risk factors associated with death within 3 years following surgery with IORT was performed with Cox proportional hazards modeling and summarized with hazard ratios (HRs) and 95% CIs.

## 3. Results

### 3.1. Patient and Disease Characteristics

Demographic and clinical characteristics of the cohort are summarized in Table 1. Eighty patients had planned radical surgery with IORT for recurrent/persistent endometrial or cervical cancer. Most patients had an ECOG PS of 0–1 (68.8%), and the median age at the time of surgery was 56.8 years. This is a heavily pretreated cohort, with 76.3% having received surgical treatment and 76.3% having received radiation prior to their presentation for disease recurrence/persistence. Eighteen patients had radiologic presence of disease within 6 months of primary treatment completion and were therefore labeled as persistent. The remaining 62 patients who later presented with recurrence had an appreciable response to primary therapy with a median disease-free interval of 20.0 months (IQR 10.0–63.1). After the diagnosis of recurrence and prior to surgery, most patients received some type of pre-surgical treatment including radiation (73.8%), chemotherapy (18.8%), and/or immunotherapy (1.3%), with some patients receiving more than one treatment. Systemic therapies used in this cohort included carboplatin, paclitaxel, cisplatin, everolimus, bevacizumab, and topotecan.

### 3.2. Surgical Outcomes

Surgical data from the cohort are summarized in Table 2. Surgical resection achieved negative margins in 72.5% of patients, and most (n = 73, 91.3%) received the planned IORT dose. Though most patients received some type of pre-operative treatment (radiation, chemotherapy, and/or immunotherapy), half (53.8%) had residual tumors larger than 3 cm in diameter prior to surgery. The majority (71.3%) had suspected sidewall involvement. Concurrent lymphadenectomy was performed in 40 patients, 20 of which included para-aortic lymphadenectomy. Microscopically positive margins were found in 12 patients (15.0%), and macroscopically positive margins were found in only 4 patients (5.0%). Supplementary Table S1 describes the seven patients who did not proceed with IORT; all were felt to have sufficient surgical excision with negative margins such that IORT was not deemed necessary by the intraoperative team. Among the patients who did receive IORT, the median dose was 1250 cGy (range 1000–2000 cGy). IORT was most frequently delivered with a circular applicator with a median diameter of 7 cm (range 4–10 cm). Eleven patients had IORT with an elliptical applicator with a median size of 6 cm × 11 cm, and one patient had IORT with a rectangular applicator measuring 8 cm × 15 cm. Two patients had more than one field treated with IORT at the time of surgery.

### 3.3. Peri-Operative Morbidity

In the 30 days after surgery, 18/80 (22.5%) were re-admitted. One patient was lost to follow up within 30 days; therefore, the remainder of the analysis focused on 79 patients. Of those 79 patients, 16 patients (20.3%) had Accordion grade 3–5 complications, and one death occurred (1.3%) (Table 2). Patients with poor ECOG PS (2–3) prior to surgery were more likely to experience grade 3+ postoperative complications compared with ECOG PS (0–1) (OR 18.00, 95% CI 1.81–178.78; *p* = 0.01), while age, smoking status, and BMI were not associated with a severe postoperative complication (Table 3). Patients who received chemotherapy and/or immunotherapy prior to surgery, after diagnosis of recurrence, were also more likely to have significant postoperative morbidity (OR 6.98, 95% CI 2.03–24.02; *p* < 0.01). Sidewall involvement was associated with grade 3+ postoperative complications (OR 8.80, 95% CI 1.09–70.86; *p* = 0.04). Other surgical factors, such as type of pelvic exenteration, margin status, and tumor diameter had no significant association with grade 3+ postoperative complications.

### 3.4. Survival Outcomes

**Figure 1:**
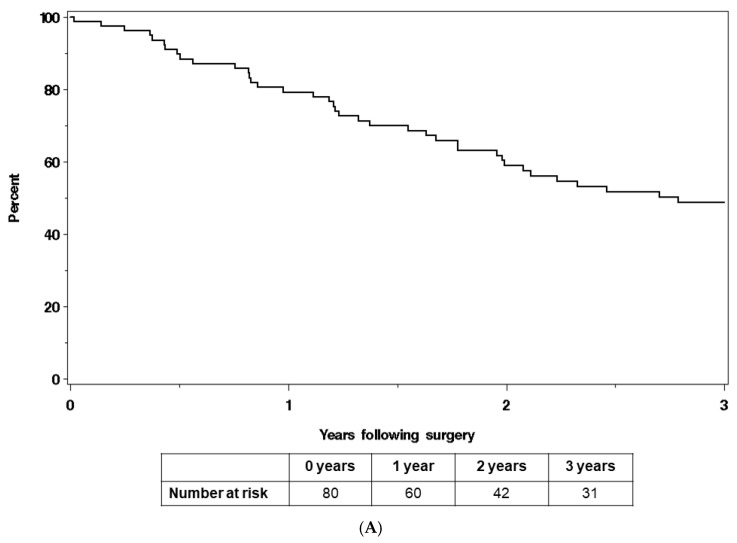

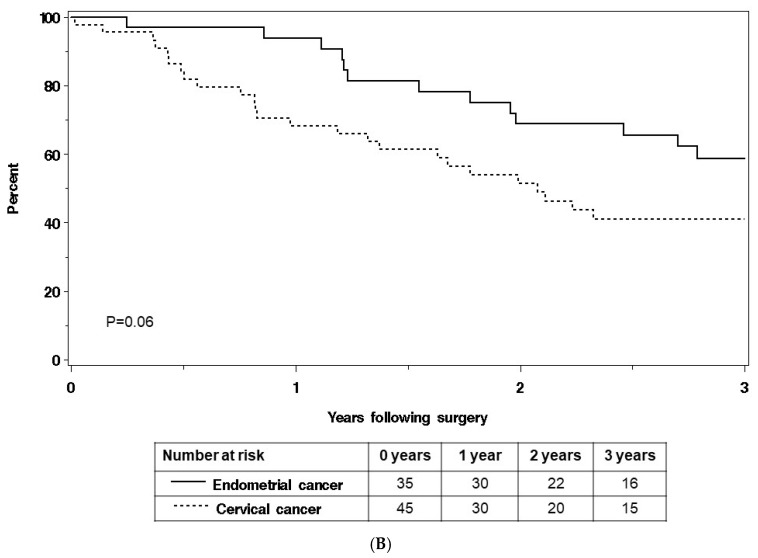
(**A**) Overall survival of endometrial and cervical cancer patients who were planned to undergo IORT for treatment of recurrent/persistent disease. (**B**) Overall survival of endometrial and cervical cancer patients who were planned to undergo IORT for treatment of recurrent/persistent disease, by disease site.

**Figure 2:**
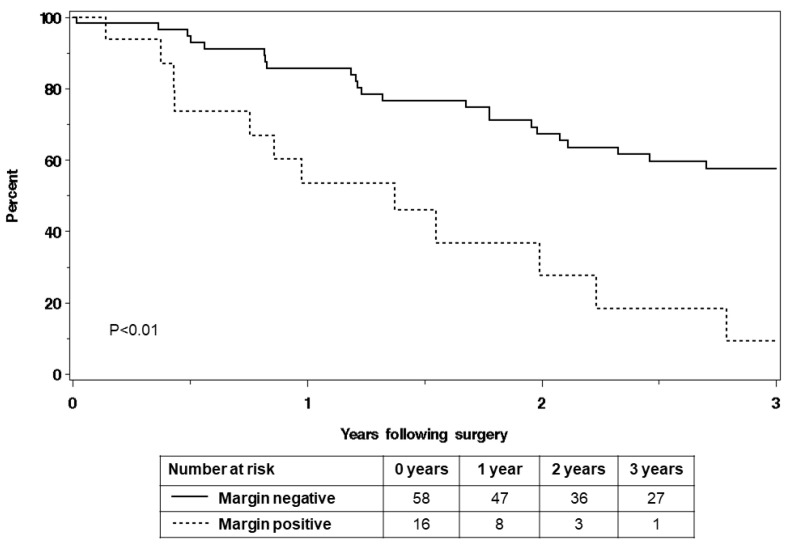
Overall survival of endometrial and cervical cancer patients who were planned to undergo IORT for treatment of recurrent/persistent disease, by resection margin.

Overall survival at three years was 48.6% (95% CI 38.3–61.6%), with a median survival of 2.8 years (Figure 1A). By primary disease site, the overall three-year survival in cervical cancer patients was 41.0% (95% CI 28.4–59.2%) and in endometrial cancer patients, it was 58.6% (95% CI 43.6–78.8%) (Figure 1B). Patients with positive resection margins (n = 16) experienced worse survival outcomes, with an overall three-year survival of 9.2% (95% CI 1.5–57.8%) compared with 57.6% (95% 45.8–72.5%) in margin-negative patients (n = 58) (Figure 2).

Table 4 presents the univariate analysis assessing factors associated with death within 3 years following surgery. Patients with recurrent cervical cancer did not have a higher risk of death within three years following surgery compared to those with recurrent endometrial cancer (HR 1.89, 95% CI 0.96–3.70; *p* = 0.06), although the power is likely limited. Use of chemotherapy and/or immunotherapy after the diagnosis of recurrence, prior to exenteration/IORT, was statistically significantly associated with an increased risk of death within three years following surgery (HR 2.34, 95% CI 1.10–4.97; *p* = 0.03). Similar to morbidity, poor ECOG PS was associated with a higher risk of death within three years (HR 8.97, 95% CI 3.25–24.71; *p* < 0.01) while smoking, age, BMI, and comorbidities were not associated. Patients requiring exenteration with IORT versus those without exenteration had worse survival, HR 2.64 (95% CI 1.25–5.59). Among the 62 patients with recurrent disease, a longer disease-free interval was associated with a lower risk of death within three years (HR 0.71 per doubling, 95% CI 0.54–0.93; *p* = 0.01). Though complete resection with negative margins was achieved in most patients, residual disease was strongly associated with death within three years compared with those with negative margins (HR 3.37, 95% CI 1.65–6.85; *p* < 0.01), while tumor diameter was not associated with death within three years.

## 4. Discussion

When cervical and endometrial cancer recur, patients are faced with likely incurable disease. While recent advances in systemic treatments such as immunotherapy, targeted agents, and antibody–drug conjugates have broadened palliative options for many patients, there are unique cases in which radical resection and IORT can prolong survival and even cure a subset of patients. In this study, we found that an appreciable survival gain is possible in patients with recurrent cervical and endometrial cancer treated with complex surgical resection and IORT, with a 3-year survival of just under 50% for the entire cohort. Cervical cancer patients had clinically shorter survival outcomes than endometrial cancer patients, with a median survival (2.1 years) less than that of the entire cohort (2.8 years), although that difference was not statistically significant. Poor ECOG PS, need for exenteration, shorter disease-free interval, use of systemic treatment prior to surgery, and positive resection margins were all associated with a greater risk of death within three years. Pursuing treatment with complex oncologic surgery with IORT is not without risks, with 20.3% of the cohort having an Accordion grade 3–5 complication and one patient death occurring within 30 days postoperatively. Clearly, patient selection is critical in balancing risks with the potential benefits.

Survival outcomes in this study are similar to those found in previous studies of the use of IORT in recurrent gynecologic malignancies. Tran et al. found a similar overall survival rate at 5 years of 47% in a mixed cohort of endometrial, cervical, fallopian tube, vaginal, and vulvar cancer patients [14]. The overall 3-year survival of 25% for patients with recurrent cervical cancer found by Barney et al. aligns with our finding that cervical cancer patients experience shorter survival than endometrial cancer patients, albeit our cohort did not have a statistically significant difference [26]. Other studies suggest similar rates of survival to our overall cohort with IORT, with Stelzer et al. reporting a 43% 5-year disease-specific survival rate [27]. Giordia et al. demonstrated similarly higher 5-year survival rates of 46% in recurrent cervical cancer patients by combining preoperative chemoradiation with surgery and IORT [28].

Systemic treatment options in recurrent endometrial and cervical cancer have made significant progress, offering substantial progression-free and overall survival benefits. Specifically, the use of pembrolizumab or dostarlimab with chemotherapy in patients with and without mismatch repair deficiency [29,30], the use of antibody–drug conjugates such as tisotumab vedotin [31], and other targeted agents, including lenvatinib, trastuzumab, and bevacizumab, now gives new hope to patients who have recurrent disease. The majority of patients with recurrent disease will use these, including some that may be able to proceed with a radical resection, such as the patients described in this study. The optimal use of systemic agents either prior to or after radical resection is not well understood and could not be assessed in this study.

We believe the risk of morbidity associated with complex oncologic surgery and IORT is worth the survival benefit in a well-selected and counseled patient cohort. Based on this study, such patients include those with good functional status (ECOG PS 0–1) as they are less likely to experience Accordion grade 3+ postoperative complications and less likely to die within three years after surgery. Similarly, less complex surgical resections not requiring total exenteration are associated with a lower risk of morbidity and mortality and would be another ideal situation for IORT use. When considering a radical resection with intraoperative radiation, the achievement of a microscopic negative margin is paramount. In this cohort, remaining positive margins following resection increased the risk of death within 3 years of surgery by more than three-fold (HR 3.37, *p* < 0.01). This highlights the need for thoughtful surgical planning to achieve such oncologic outcomes. Selecting the patients best suited for complex resection and IORT requires acknowledgment of the realities of this patient population, the morbidity risks and survival benefits of this treatment modality, and open discussion with each patient. Furthermore, assembling appropriate teams with proper preoperative imaging to achieve negative margins and apply IORT thoughtfully is extremely important. We have assembled a focused complex oncologic resection team for recurrent cases requiring IORT, such as those described in this study. This type of multi-disciplinary approach is critical to achieve good patient outcomes.

The strengths of this study include the extensive experience at these three institutions with a large amount of data. The limitations are those inherent to a retrospective study, including our inability to understand all the reasoning and tumor characteristics, pre- and intra-operative, that led to some management decisions. This group of patients is extremely heterogeneous, and an understanding of their complex cancer journeys cannot be achieved through a chart review. We are unable to report on any patient-reported outcomes.

## 5. Conclusions

In summary, the use of IORT with surgical resection can achieve 3-year survival rates of almost 50% in recurrent/persistent cervical and endometrial cancer patients, with endometrial cancer patients having better survival outcomes than cervical cancer patients. Poor functional status, more complex surgical resections, and having received chemotherapy and/or immunotherapy for the recurrence increased risk of morbidity and/or mortality; however, these characteristics are often unavoidable realities of patients with recurrent cancers and must be considered in the context of the individual patient and their wishes for treatment. Complete surgical resection with negative margins remains paramount in achieving improved survival outcomes. At our institution, we have developed a multi-disciplinary approach to these patients including involvement with multiple surgical teams, supportive care teams, including palliative care and social work and psycho-oncology, and extensive patient education, including patient mentorship and specific materials for this cohort of patients. Given the rarity of this type of radical oncologic excision, we believe that proper planning and referral to the few institutions with IORT experience is paramount for good outcomes. While we can cure some patients, there is still a great deal more work to be accomplished to improve oncologic and quality-of-life outcomes.

## Figures and Tables

**Table 1:** Characteristics of patients with planned IORT for recurrence.

Characteristic	Total N = 80
Age at surgery (years), mean (SD)	56.8 (13.7)
Tumor site	
Cervical	45 (56.3)
Endometrial	35 (43.8)
BMI (kg/m^2^), mean (SD)	26.5 (6.8)
ECOG performance status	
0–1	55 (68.8)
2–3	5 (6.3)
Unknown/not documented	20 (25.0)
Smoking status	
Never smoked	47 (58.8)
Former/current smoker	33 (41.3)
Comorbidities *	
0	27 (33.8)
1	24 (30.0)
2	15 (18.8)
3+	14 (17.5)
Previous surgical treatment of the disease	61 (76.3)
Previous radiation (EBRT and/or VBT)	61 (76.3)
Previous systemic chemotherapy	21 (26.3)
Timing of disease at presentation for pelvic exenteration	
Persistent	18 (22.5)
Recurrent	62 (77.5)
Disease-free interval (months), median (IQR) ^†^	20.0 (10.0, 63.1)

Abbreviations: BMI, body mass index; ECOG, Eastern Cooperative Oncology Group; IQR, interquartile range; SD, standard deviation; EBRT, external beam radiation therapy; VBT, vaginal brachytherapy. Results are presented as N (%) unless otherwise specified. * Comorbidities included history of myocardial infarction, congestive heart failure, percutaneous coronary intervention (PCI) or prior cardiac surgery, hypertension requiring medication, diabetes mellitus, chronic obstructive pulmonary disease (COPD), transient ischemic attack (TIA) or cerebrovascular accident (CVA), venous thromboembolism (VTE), depression, renal failure, liver disease, autoimmune disease, rheumatologic disease, or hyperlipidemia. ^†^ Among the 62 patients who presented with recurrent disease (31 endometrial, 31 cervical), excluding those with persistent disease.

**Table 2:** Perioperative characteristics.

Characteristic	Total N = 80
Exenteration performed	
No	31 (38.8)
Yes	49 (61.3)
Type of exenteration	
N/A	31 (38.8)
Total	28 (35.0)
Anterior	11 (13.8)
Posterior	10 (12.5)
Suspected side wall involvement	57 (71.3)
Number of surgical services involved in the case *	
1	35 (43.8)
2	24 (30.0)
3	18 (22.5)
4	3 (3.8)
Tumor diameter	
≤3 cm	11 (13.8)
>3 cm	43 (53.8)
Unknown	26 (32.5)
Resection margin	
Negative	58 (72.5)
Positive	16 (20.0)
Not documented	6 (7.5)
Estimated blood loss (mL), median (IQR)	750 (375, 1600)
Intraoperative radiation dose (cGy), median (IQR)	1250 (1000, 1500)
ICU stay	17 (21.3)
Readmission within 30 days	18 (22.5)
Accordion grade complication	
0–2	62 (77.5)
3	8 (10.0)
4	7 (8.8)
5	1 (1.3)
6 (death)	1 (1.3)
Unknown (lost to follow-up)	1 (1.3)

Abbreviations: ICU, intensive care unit; IQR, interquartile range. * Surgical services other than gynecologic surgery and radiation oncology include plastic surgery, urology, hepatobiliary surgery, orthopedics, and vascular surgery.

**Table 3:** Factors associated with Accordion grade 3+ complications within 30 days postoperative.

Characteristic	N of Patients with Accordion Grade 3+ Complications	Univariate OR (95% CI)	*p*
Age at time of surgery (years)	17	1.13 (0.76, 1.69) *	0.55
Primary site			0.47
Endometrial (N = 34)	6	Reference	
Cervical (N = 45)	11	1.51 (0.50, 4.60)	
BMI (kg/m^2^)	17	0.66 (0.39, 1.13) *	0.13
ECOG performance status			0.04
0–1 (N = 55)	10	Reference	
2–3 (N = 5)	4	18.00 (1.81, 178.78)	
Unknown/not documented (N = 19)	3	0.84 (0.21, 3.46)	
Smoking status			0.09
Never smoked (N = 47)	7	Reference	
Former/current smoker (N = 32)	10	2.60 (0.87, 7.78)	
Comorbidities *			0.99
0 (N = 27)	6	Reference	
1 (N = 23)	5	0.97 (0.25, 3.73)	
2 (N = 15)	3	0.88 (0.18, 4.15)	
3+ (N = 14)	3	0.96 (0.20, 4.57)	
Any treatment prior to surgery, after diagnosis of recurrence (N = 64)	16	4.67 (0.57, 38.35)	0.15
Chemotherapy and/or immunotherapy prior to surgery, after diagnosis of recurrence (N = 15)	8	6.98 (2.03, 24.02)	<0.01
Radiation prior to surgery, after diagnosis of recurrence (N = 58)	12	0.84 (0.25, 2.74)	0.77
Exenteration performed (N = 49)	13	2.35 (0.69, 8.02)	0.17
Type of exenteration			0.49
N/A (N = 30)	4	Reference	
Total (N = 28)	8	2.60 (0.69, 9.87)	
Anterior (N = 11)	2	1.44 (0.23, 9.27)	
Posterior (N = 10)	3	2.79 (0.50, 15.46)	
Suspected side wall involvement (N = 56)	16	8.80 (1.09, 70.86)	0.04
Tumor diameter			0.38
≤3 cm (N = 11)	4	Reference	
>3 cm (N = 42)	9	0.48 (0.11, 2.00)	
Unknown (N = 26)	4	0.32 (0.06, 1.62)	
Resection margin			0.15
Negative (N = 57)	9	Reference	
Positive (N = 16)	6	3.20 (0.93, 11.03)	
Not documented (N = 6)	2	2.67 (0.42, 16.80)	
Estimated blood loss (mL)	16	1.37 (0.92, 2.04) *	0.12

Abbreviations: BMI, body mass index; CI, confidence interval; ECOG, Eastern Cooperative Oncology Group; OR, odds ratio; SD, standard deviation. * Odds ratio per 10-year increase in age, per 5 kg/m^2^ increase in BMI, and per doubling in estimated blood loss. * Comorbidities included history of myocardial infarction, congestive heart failure, percutaneous coronary intervention (PCI) or prior cardiac surgery, hypertension requiring medication, diabetes mellitus, chronic obstructive pulmonary disease (COPD), transient ischemic attack (TIA) or cerebrovascular accident (CVA), venous thromboembolism (VTE), depression, renal failure, liver disease, autoimmune disease, rheumatologic disease, or hyperlipidemia.

**Table 4:** Factors associated with death within three years of surgery.

Characteristic	N with Event	Univariate HR (95% CI)	*p*
Age at exenteration (years)	38	0.82 (0.65, 1.04) *	0.10
Primary site			0.06
Endometrial (N = 35)	13	Reference	
Cervical (N = 45)	25	1.89 (0.96, 3.70)	
BMI (kg/m^2^)	38	0.99 (0.75, 1.31) *	0.95
ECOG performance status			<0.01
0–1 (N = 55)	22	Reference	
2–3 (N = 5)	5	8.97 (3.25, 24.71)	
Unknown/not documented (N = 20)	11	1.25 (0.61, 2.59)	
Disease-free interval (months)	32	0.71 (0.54, 0.93) *^^^	0.01
Smoking status			0.98
Never smoked (N = 47)	22	Reference	
Former/current smoker (N = 33)	16	1.01 (0.53, 1.92)	
Comorbidities *			0.61
0 (N = 27)	11	Reference	
1 (N = 24)	14	1.52 (0.69, 3.35)	
2 (N = 15)	6	0.86 (0.32, 2.34)	
3+ (N = 14)	7	1.09 (0.42, 2.82)	
Any treatment prior to exenteration/IORT, after diagnosis of recurrence (N = 65)	31	1.09 (0.48, 2.48)	0.84
Chemotherapy and/or immunotherapy prior to exenteration/IORT, after diagnosis of recurrence (N = 15)	9	2.34 (1.10, 4.97)	0.03
Radiation prior to exenteration/IORT, after diagnosis of recurrence (N = 59)	27	0.74 (0.37, 1.50)	0.40
Exenteration performed (N = 49)	29	2.64 (1.25, 5.59)	0.01
Tumor diameter			0.32
≤3 cm (N = 11)	7	Reference	
>3 cm (N = 43)	18	0.51 (0.21, 1.22)	
Unknown (N = 26)	13	0.65 (0.26, 1.62)	
Resection margin			<0.01
Negative (N = 58)	23	Reference	
Positive (N = 16)	12	3.37 (1.65, 6.85)	
Not documented (N = 6)	3	1.41 (0.42, 4.68)	

Abbreviations: BMI, body mass index; CI, confidence interval; ECOG, Eastern Cooperative Oncology Group; HR, hazard ratio; IORT, intraoperative radiation therapy. * Hazard ratio per 10-year increase in age, per 5 kg/m^2^ increase in BMI, and per doubling in disease-free interval. ^^^ Among the 62 patients who present with recurrent disease.

## Data Availability

De-identified data will be made available upon request to the corresponding author.

## Supplementary Materials

The following supporting information can be downloaded at: https://www.mdpi.com/article/10.3390/cancers16213628/s1, Table S1: Patients with planned IORT who did not receive intraoperative radiation therapy.

## References

[1] Siegel R.L., Giaquinto A.N., Jemal A. (2024). Cancer Statistics, 2024. CA Cancer J. Clin..

[2] Rütten H., Verhoef C., van Weelden W.J., Smits A., Dhanis J., Ottevanger N., Pijnenborg J.M.A. (2021). Recurrent Endometrial Cancer: Local and Systemic Treatment Options. Cancers.

[3] Shen Z., Qu A., Jiang P., Jiang Y., Sun H., Wang J. (2022). Re-Irradiation for Recurrent Cervical Cancer: A State-of-the-Art Review. Curr. Oncol..

[4] Kasamatsu T., Onda T., Yamada T., Tsunematsu R. (2005). Clinical Aspects and Prognosis of Pelvic Recurrence of Cervical Carcinoma. Int. J. Gynecol. Obstet..

[5] de Boer S.M., Powell M.E., Mileshkin L., Katsaros D., Bessette P., Haie-Meder C., Ottevanger P.B., Ledermann J.A., Khaw P., D’Amico R. (2019). Adjuvant Chemoradiotherapy versus Radiotherapy Alone in Women with High-Risk Endometrial Cancer (PORTEC-3): Patterns of Recurrence and Post-Hoc Survival Analysis of a Randomised Phase 3 Trial. Lancet Oncol..

[6] Arden J.D., Gruner M.F., Vu C.C., Marvin K., Ye H., Nandalur S.R., Al-Wahab Z., Gadzinski J., Rakowski J.A., Field J. (2020). Outcomes After Salvage Radiation Therapy for Recurrent Endometrial Cancer in Patients with No Prior Adjuvant Therapy: An Institutional Review. Adv. Radiat. Oncol..

[7] Kim H.J., Chang J.S., Koom W.S., Lee K.C., Kim G.E., Kim Y.B. (2018). Radiotherapy Is a Safe and Effective Salvage Treatment for Recurrent Cervical Cancer. Gynecol. Oncol..

[8] Hardarson H.A., Heidemann L.N., Depont Christensen R., Mogensen O., Jochumsen K.M. (2015). Vaginal Vault Recurrences of Endometrial Cancer in Non-Irradiated Patients—Radiotherapy or Surgery. Gynecol. Oncol. Rep..

[9] Francis S.R., Ager B.J., Do O.A., Huang Y.H.J., Soisson A.P., Dodson M.K., Werner T.L., Sause W.T., Grant J.D., Gaffney D.K. (2019). Recurrent Early Stage Endometrial Cancer: Patterns of Recurrence and Results of Salvage Therapy. Gynecol. Oncol..

[10] Restaino S., Dinoi G., La Fera E., Gui B., Cappuccio S., Campitelli M., Vizzielli G., Scambia G., Fanfani F. (2022). Recurrent Endometrial Cancer: Which Is the Best Treatment? Systematic Review of the Literature. Cancers.

[11] Dhanis J., Blake D., Rundle S., Pijnenborg J.M.A., Smits A. (2022). Cytoreductive Surgery in Recurrent Endometrial Cancer: A New Paradigm for Surgical Management?. Surg. Oncol..

[12] Chiva L.M., Lapuente F., González-Cortijo L., González-Martín A., Rojo A., García J.F., Carballo N. (2008). Surgical Treatment of Recurrent Cervical Cancer: State of the Art and New Achievements. Gynecol. Oncol..

[13] Awtrey C.S., Cadungog M.G., Leitao M.M., Alektiar K.M., Aghajanian C., Hummer A.J., Barakat R.R., Chi D.S. (2006). Surgical Resection of Recurrent Endometrial Carcinoma. Gynecol. Oncol..

[14] Tran P.T., Su Z., Hara W., Husain A., Teng N., Kapp D.S. (2007). Long-Term Survivors Using Intraoperative Radiotherapy for Recurrent Gynecologic Malignancies. Int. J. Radiat. Oncol. Biol. Phys..

[15] Garton G.R., Gunderson L.L., Webb M.J., Wilson T.O., Cha S.S., Podratz K.C. (1997). Intraoperative Radiation Therapy in Gynecologic Cancer: Update of the Experience at a Single Institution. Int. J. Radiat. Oncol. Biol. Phys..

[16] Backes F.J., Billingsley C.C., Martin D.D., Tierney B.J., Eisenhauer E.L., Cohn D.E., O’Malley D.M., Salani R., Copeland L.J., Fowler J.M. (2014). Does Intra-Operative Radiation at the Time of Pelvic Exenteration Improve Survival for Patients with Recurrent, Previously Irradiated Cervical, Vaginal, or Vulvar Cancer?. Gynecol. Oncol..

[17] Elashwah A., Alsuhaibani A., Alzahrani A., Azzam A.Z., Moftah B., Breakeit M., Hussain M., Mahmood R., ALramahi S., Hassan Z. (2023). The Use of Intraoperative Radiation Therapy (IORT) in Multimodality Management of Cancer Patients: A Single Institution Experience. J. Gastrointest. Cancer.

[18] Biete A., Oses G. (2018). Intraoperative Radiation Therapy in Uterine Cervical Cancer: A Review. Rep. Pract. Oncol. Radiother..

[19] Krempien R., Roeder F. (2017). Intraoperative Radiation Therapy (IORT) in Pancreatic Cancer. Radiat. Oncol..

[20] Krengli M., Pisani C., Deantonio L., Surico D., Volpe A., Surico N., Terrone C. (2017). Intraoperative Radiotherapy in Gynaecological and Genito-Urinary Malignancies: Focus on Endometrial, Cervical, Renal, Bladder and Prostate Cancers. Radiat. Oncol..

[21] Ebad Ali S.M., Abbasi A.N., Zahoor N. (2022). Outcomes of Intraoperative Radiotherapy (IORT) for Spinal Tumours. J. Ayub Med. Coll. Abbottabad Pak..

[22] Llewelyn M., Taylor A. (2017). Re-Irradiation of Cervical and Endometrial Cancer. Curr. Opin. Oncol..

[23] Backes F.J., Martin D.D. (2015). Intraoperative Radiation Therapy (IORT) for Gynecologic Malignancies. Gynecol. Oncol..

[24] Dowdy S.C., Mariani A., Cliby W.A., Haddock M.G., Petersen I.A., Sim F.H., Podratz K.C. (2006). Radical Pelvic Resection and Intraoperative Radiation Therapy for Recurrent Endometrial Cancer: Technique and Analysis of Outcomes. Gynecol. Oncol..

[25] Strasberg S.M., Linehan D.C., Hawkins W.G. (2009). The Accordion Severity Grading System of Surgical Complications. Ann. Surg..

[26] Barney B.M., Petersen I.A., Dowdy S.C., Bakkum-Gamez J.N., Klein K.A., Haddock M.G. (2013). Intraoperative Electron Beam Radiotherapy (IOERT) in the Management of Locally Advanced or Recurrent Cervical Cancer. Radiat. Oncol..

[27] Stelzer K.J., Koh W.J., Greer B.E., Cain J.M., Tamimi H.K., Figge D.C., Goff B.A., Griffin T.W. (1995). The Use of Intraoperative Radiation Therapy in Radical Salvage for Recurrent Cervical Cancer: Outcome and Toxicity. Am. J. Obstet. Gynecol..

[28] Giorda G., Boz G., Gadducci A., Lucia E., De Piero G., De Paoli A., Innocente R., Trov M., Sorio R., Campagnutta E. (2011). Multimodality Approach in Extra Cervical Locally Advanced Cervical Cancer: Chemoradiation, Surgery and Intra-Operative Radiation Therapy. A Phase II Trial. Eur. J. Surg. Oncol..

[29] Mirza M.R., Chase D.M., Slomovitz B.M., dePont Christensen R., Novák Z., Black D., Gilbert L., Sharma S., Valabrega G., Landrum L.M. (2023). Dostarlimab for Primary Advanced or Recurrent Endometrial Cancer. N. Engl. J. Med..

[30] Eskander R.N., Sill M.W., Beffa L., Moore R.G., Hope J.M., Musa F.B., Mannel R., Shahin M.S., Cantuaria G.H., Girda E. (2023). Pembrolizumab plus Chemotherapy in Advanced Endometrial Cancer. N. Engl. J. Med..

[31] Coleman R.L., Lorusso D., Gennigens C., González-Martín A., Randall L., Cibula D., Lund B., Woelber L., Pignata S., Forget F. (2021). Efficacy and Safety of Tisotumab Vedotin in Previously Treated Recurrent or Metastatic Cervical Cancer (InnovaTV 204/GOG-3023/ENGOT-Cx6): A Multicentre, Open-Label, Single-Arm, Phase 2 Study. Lancet Oncol..

