# Supplementary material for: Intraoperative Radiation Therapy for Recurrent Cervical and Endometrial Cancer: Predicting Morbidity and Mortality in a Contemporary Cohort
Source: Cancers (Basel). 2024 Oct 28;16(21):3628. doi: 10.3390/cancers16213628 (PMC11545734; doi:10.3390/cancers16213628)
Supplement: Supplementary file 1 [file cancers-16-03628-s001.zip › cancers-3226298-supplementary.pdf]

Supplementary Materials

Supplementary Table S1. Patients with planned IORT who did not receive intraoperative radiation therapy.

Age	Disease site	Preoperative Radiation dose	Surgery Completed	Reason for not proceeding with IORT
33	Cervix	50 Gy pre-IORT	Pelvic exenteration (total)	Resection felt to be sufficient w/ negative margins
63	Cervix	49.5 Gy pre-IORT<br>56.25 Gy EBRT<br>30 Gy VBT	Resection of tumor and paraaortic lymph nodes	Resection felt to be sufficient w/ negative margins
71	Endometrium	30 Gy pre-IORT<br>45 Gy EBRT<br>21 Gy VBT	Pelvic exenteration (total)	Resection felt to be sufficient w/ negative margins; concerns of prior treatment effect
69	Endometrium	55 Gy pre-IORT	Left pelvic sidewall resection w/ descending colostomy	Resection felt to be sufficient w/ negative margins
53	Endometrium	55 Gy pre-IORT<br>45 Gy EBRT<br>5 Gy VBT	Paraaortic lymph node resection	Dissected lymph nodes negative for viable tumor
44	Cervix	50 Gy pre-IORT<br>45 Gy EBRT<br>6 Gy VBT	Paraaortic lymph node and right common iliac lymph node resection	Dissected lymph nodes negative for residual disease
57	Cervix	45 Gy EBRT<br>9 Gy VBT	Pelvic exenteration (posterior)	Resection felt to be sufficient w/ negative margins; concerns of prior treatment effect

Abbreviations: IORT; intraoperative radiation therapy; EBRT, external beam radiation therapy; VBT, vaginal brachytherapy.
